# Supplementary material for: Artificial dragonfly algorithm in the Hopfield neural network for optimal Exact Boolean k satisfiability representation
Source: PLoS One. 2023 Sep 25;18(9):e0286874. doi: 10.1371/journal.pone.0286874 (PMC10519610; doi:10.1371/journal.pone.0286874)
Supplement: Attachment — Submitted filename: Response to reviewers.docx [file pone.0286874.s001.docx]

*First of all, I would like to thank the reviewers for their constructive comments and recommendations. The comments and reviewers are mean the world to us to improve the manuscript. As promised, we have made revisions and amendments accordingly. The response to reviewers comment are enclosed below.*

**Response to Reviewer(s)' Comments**

Thank you for the constructive comments, we have update our paper better as requested by reviewers.

----Reviewer #1:---

***Reviewer(s)' Comments to Author***

1. Is there any particular reason why the entire text is highlighted in yellow? It made reading very hard.

**Response to Reviewer(s)' Comments**

It is the revised version. We are sorry about that. Thank you very much

***Reviewer(s)' Comments to Author***

2. Avoid using acronyms in the abstract.

**Response to Reviewer(s)' Comments**

Thank you very much for this comment. All acronyms has been removed accordingly.

***Reviewer(s)' Comments to Author***

3. Introduction is too long. Separate it into Introduction and Literature review, two distinctive sections.

**Response to Reviewer(s)' Comments**

This section has been separated accordingly.

***Reviewer(s)' Comments to Author***

4. Paragraphs are too long as well, consider breaking them in smaller units.

**Response to Reviewer(s)' Comments**

This section has been revised accordingly. Thank you very much for comments

***Reviewer(s)' Comments to Author***

5. State the research question clearly in the Introduction.

**Response to Reviewer(s)' Comments**

Thank you very much for this comments. We have addeded research question accordongly

***Reviewer(s)' Comments to Author***

6. List the main contributions clearly in the Introduction (they are present, but should be emphasized more, for example like a list).

**Response to Reviewer(s)' Comments**

The main contributions is clearly stated. Thank you very much for this comments

***Reviewer(s)' Comments to Author***

7. The literature review could be expanded, consider adding the following references that deal with dragonfly algorithm, and also ANN optimization by metaheuristics:
https://www.sciencedirect.com/science/article/abs/pii/S2210537922000506
https://www.sciencedirect.com/science/article/abs/pii/S0003682X21002152
https://link.springer.com/chapter/10.1007/978-981-15-8530-2_63
https://link.springer.com/article/10.1007/s00521-022-06946-7
<https://www.mdpi.com/1996-1073/16/3/1434>

**Response to Reviewer(s)' Comments**

Thank you very much. We have cited theses articles accordingly

***Reviewer(s)' Comments to Author***

8. Make sure that each parameter in every equation has been explained in the text.

**Response to Reviewer(s)' Comments**

All parameters have been explained accordingly.

***Reviewer(s)' Comments to Author***

9. Overall quality of figures should be improved - especially flowchart (fig. 2).

**Response to Reviewer(s)' Comments**

Thank you very much for this comment. We have replotted all figures with high resolutions.

***Reviewer(s)' Comments to Author***

10. Discuss more why you have selected dragonfly - as there are numerous other metaheuristics algorithms.

**Response to Reviewer(s)' Comments**

Thank you very much. There is no specifi reason for this selction only to explored the performance of dragonfly

***Reviewer(s)' Comments to Author***

11. Discuss the limitations of the proposed method in more details.

**Response to Reviewer(s)' Comments**

We have added discussion accordingly.

***Reviewer(s)' Comments to Author***

12. Elaborate more on the simulation setup - also give the results in tabular form (not only by graphs and charts).

**Response to Reviewer(s)' Comments**

Thank you for the constructive comments, we added table to show the best result as requste by the reviewer.

**----Reviewer #2: ----**

***Reviewer(s)' Comments to Author***

In this paper, a hybrid computational method combining the artificial dragonfly algorithm (ADA) with the Hopfield neural network techniques for optimal Exact k Satisfiability representation is presented.

***Reviewer(s)' Comments to Author***

Several issues in this paper need to be addressed before publication:
The paper requires some reorganization. In the Introduction section, it is important to provide a brief overview of the topic addressed in the paper, along with a concise description of the main contributions, which are currently listed but need to be explained in greater detail. Additionally, a new section entitled "Related Work" should be added to the paper, which will include a description of previously used methodologies and systems that are currently part of the Introduction section.

**Response to Reviewer(s)' Comments**

Thank you for the constructive comments, we added the related work at section of literature review as requste by the reviewer.

***Reviewer(s)' Comments to Author***

Furthermore, it is essential to enhance the quality of Figure 2. Additionally, Figure 6 contains typos that need to be corrected.

**Response to Reviewer(s)' Comments**

Thank you for the constructive comments, we refigure all figures.

***Reviewer(s)' Comments to Author***

Regarding the evaluation, it is important to note that simulated datasets were used to establish the Exact kSAT logical clauses. However, further explanation is required to fully understand this process. Therefore, additional effort is needed to test and prove the set hypotheses on the available datasets.

**Response to Reviewer(s)' Comments**

in this work we use simulated data, Simulated data is a randomly generated data by our program during the simulation. The generated data considers the binary values with the structure based on the number of clauses defined by the researchers. Thus, the simulated data is commonly used to authenticate the performance our proposed networks in discriminating the solutions, whether it is an optimal or suboptimal solution. Therefore, the database is massive, and it covers a more diverse search space. The concept of simulated data in SAT logic with ANN has been initially coined in the work of Saratha, S. (2010) in HNN development and Abdulhabib et al. (2021).
