# Supplementary material for: Artificial dragonfly algorithm in the Hopfield neural network for optimal Exact Boolean k satisfiability representation
Source: PLoS One. 2023 Sep 25;18(9):e0286874. doi: 10.1371/journal.pone.0286874 (PMC10519610; doi:10.1371/journal.pone.0286874)
Supplement: Attachment — Submitted filename: Response to reviewers RV 11.docx [file pone.0286874.s002.docx]

*First of all, I would like to thank the reviewers for their constructive comments and recommendations. The comments and reviewers are mean the world to us to improve the manuscript. As promised, we have made revisions and amendments accordingly. The response to reviewers comment are enclosed below.*

**Response to Reviewer(s)' Comments**

Thank you for the constructive comments, we have update our paper better as requested by reviewers.

**---- ACADEMIC EDITOR :---**

***Academic (s)' Comments to Author***

- Figure quality should be improved, e.g. x-axis titles are too large and they should be resized.

Response To Academic Editor(S)' Comments

The quality of figures have been improve accordingly. Thank you very for this comment.

***Academic (s)' Comments to Author***

- There are many inconsistencies in titles numbering, e.g. "1. Materials and Methods 2.1 Exact k Satisfiability of a Boolean Formula".

**Response To Academic Editor(S)' Comments**

It is sincerely regarrted. All inconsistenies have been checked and corrected accoringly

***Academic (s)' Comments to Author***

- Please make sure that all notations in expressions are defined.

**Response To Academic Editor(S)' Comments**

All notations in expressions have been defined accordingly.

***Academic (s)' Comments to Author***

- Please conduct appropriate statistical analysis.

**Response To Academic Editor(S)' Comments**

Thank you very much for this comment. An appropriate statistical analysis have been carried out accordingly.

***Academic (s)' Comments to Author***

- Conclusion should be extended to include practical and theoretical limitations of proposed study.

**Response To Academic Editor(S)' Comments**

Thank you very much. Some of the practical and theoretical limitations of proposed study have been highlighlited accordingly

***Academic (s)' Comments to Author***

- Please make sure that you ad here with all PLOS ONE policies

**Response To Academic Editor(S)' Comments**

It is the revised version. We are sorry about that. Thank you very much
